# Chromatographic Determination of the Mycotoxin Patulin in 219 Chinese Tea Samples and Implications for Human Health

**DOI:** 10.3390/molecules27092852

**Published:** 2022-04-29

**Authors:** Hai Li, Candi Liu, Shurong Luo, Sijie Zhu, Shan Tang, Huimei Zeng, Yu Qin, Ming Ma, Dong Zeng, Teris A. van Beek, Hui Wang, Bo Chen

**Affiliations:** 1 Key Laboratory of Phytochemical R&D of Hunan Province, Key Laboratory of Chemical Biology & Traditional Chinese Medicine Research of Ministry of Education, Hunan Normal University, Changsha 410081, China; 202120122346@hunnu.edu.cn (H.L.); ayaneruqaq@foxmail.com (C.L.); 202120122465@hunnu.edu.cn (S.L.); 202120122423@hunnu.edu.cn (S.Z.); 202120122374@hunnu.edu.cn (S.T.); 202120122311@hunnu.edu.cn (H.Z.); 202120122368@hunnu.edu.cn (Y.Q.); mingma@hunnu.edu.cn (M.M.); 2 Hunan Provincial Center for Disease Control and Prevention, Changsha 410005, China; 3 Laboratory of Organic Chemistry, Wageningen University, Stippeneng 4, 6708 WE Wageningen, The Netherlands; 4 Changsha Institute for Food and Drug Control, National Quality Supervision and Inspection Center of Liquor Products (Hunan), Changsha 410013, China; wanghuei158@163.com

**Keywords:** patulin, tea, HPLC−DAD, GC−MS, mycotoxin detection

## Abstract

Patulin (PAT) is a mycotoxin, with several acute, chronic, and cellular level toxic effects, produced by various fungi. A limit for PAT in food of has been set by authorities to guarantee food safety. Research on PAT in tea has been very limited although tea is the second largest beverage in the world. In this paper, HPLC−DAD and GC−MS methods for analysis of PAT in different tea products, such as non-fermented (green tea), partially fermented (oolong tea, white tea, yellow tea), completely fermented (black tea), and post-fermented (dark tea and Pu-erh tea) teas were developed. The methods showed good selectivity with regard to tea pigments and 5-hydroxymethylfurfural (5-HMF) and a recovery of 90–102% for PAT at a 10–100 ppb spiking level. Limit of detection (LOD) and limit of quantification (LOQ) in tea were 1.5 ng/g and 5.0 ng/g for HPLC−UV, and 0.25 ng/g and 0.83 ng/g for GC−MS. HPLC was simpler and more robust, while GC−MS showed higher sensitivity and selectivity. GC−MS was used to validate the HPLC−UV method and prove its accuracy. The PAT content of 219 Chinese tea samples was investigated. Most tea samples contained less than 10 ng/g, ten more than 10 ng/g and two more than 50 ng/g. The results imply that tea products in China are safe with regard to their PAT content. Even an extreme daily consumption of 25 g of the tea with the highest PAT content (124 ng/g), translates to an intake of only 3 μg/person/day, which is still an order of magnitude below the maximum allowed daily intake of 30 µg for an adult.

## 1. Introduction

**Figure 1:**
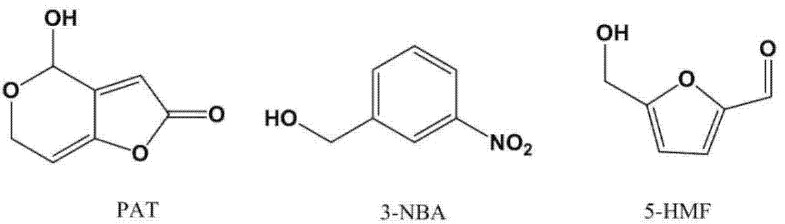
Chemical structure of patulin (PAT), 3-nitrobenzyl alcohol (3-NBA), and 5-hydroxymethylfurfural (5-HMF).

Mycotoxins are naturally occurring, toxic chemicals produced by fungi. Foods and fodder infected by such fungi pose serious risks for human and animal health. Many mycotoxins have been identified, e.g., aflatoxins, ochratoxin A, fumonisins, patulin, moniliformin, sterigmatocystin, tricothecenes and zearalenone, and their presence in foods and fodder is undesirable [1]. Patulin (PAT) (Figure 1) is a mycotoxin produced by a number of species of Aspergillus and Penicillium and a group 3 carcinogen. It has neurological, gastrointestinal and immunological effects, and is considered as a possible genotoxic compound by the WHO [2,3,4,5]. Fruits and vegetables are the most common foods in which PAT is encountered [6]. Apart from fruits and fruit-based products, PAT can also be found in cereals, cereal products [7], and seafoods such as shellfish [8].

For safety reasons, the FAO/WHO Joint Expert Committee on Food Additives and Contaminants (JECFA) implemented a provisional maximum tolerable daily intake (PMTDI) of 0.4 μg/kg body weight/day for PAT, which equals 30 µg/day for a 75 kg adult. The maximum acceptable level of PAT in apple juice is set at 50 μg/L for adults and at 10 μg/L in apple-based foods for infants and babies [9].

In addition to apples and apple-based products, PAT has also been found in other fruit commodities at too high levels, e.g., dried figs and dried longans in China (65% and 91% positive samples, average PAT concentrations of 131 μg/kg and 75 μg/kg, and maximum concentrations of 277 μg/kg and 194 μg/kg, respectively) [10]. Unfortunately, no information on PAT exposure assessment was available for the food with higher PAT levels, and risk assessments were not included.

Tea, produced from the leaves of *Camellia sinensis*, is the second most frequently consumed beverage in the world [11,12]. Average daily consumption varies significantly per country: Turkey has the highest consumption (8.65 g/person/day) while in some countries, tea consumption is negligible. In China, the average use is 1.55 g/person/day [13]. Tea products come in different forms such as non-fermented tea (green tea), partially fermented tea (oolong tea, yellow tea, white tea), completely fermented tea (black tea), and post-fermented tea (dark tea and Pu-erh tea). The distinction is based on the processing methods with each kind having a different degree of fermentation [14]. As microbial fermentation is essential for flavor formation during processing, contamination by harmful microorganisms is possible and could affect the safety of tea products [15,16,17]. A further distinction of tea products is based on aging (so-called “new” tea and “old” tea). Also, during this ageing process, tea may become contaminated with mycotoxins [18]. A number of mycotoxins such as aflatoxin B1, deoxynivalenol and ochratoxin A have been reported in different tea products [9,19]. 

Therefore, more attention is being paid to the occurrence of those mycotoxins in tea products and concomitant safety issues [20,21,22]. However, the research on PAT in tea has been limited even though PAT contamination in foods is a worldwide problem. In a function and safety study of *Penicillium oxalicum* during the fermentative process of Pu-erh tea, a PAT level of 12.6 μg/kg was found in one tea sample [23]. In a study on the microbiome and metabolites in fermented Pu-erh tea, high PAT levels of 1169 μg/kg in raw samples and 915 μg/kg in ripened samples were found [24]. The authors suggested that this may have been caused by yet unknown PAT-producing fungal species in these tea products. Due to the enormous variation of reported PAT levels in tea, it is currently unclear whether the occurrence of PAT in tea poses a problem to human health. There is also a lack of knowledge on the influence of the fermentation and aging processes of tea on PAT concentrations. Establishing the PAT concentration in more than 200 Chinese tea samples with varying degrees of fermentation to assess the health risks of PAT ingestion via tea consumption constituted the main aim of this paper.

A prerequisite for the determination of PAT in tea is an accurate validated analytical method. Different chromatography-based methods such as GC−MS, HPLC with UV detection at 276 nm, and HPLC-tandem mass spectrometry (HPLC−MS/MS), have been employed [9,25,26,27,28,29,30,31,32,33,34,35,36]. A review on the analysis of patulin has been recently published [37]. However, these methods have focused on fruits such as apples and fruit-derived products. The validation of analytical methods for PAT in tea or products thereof has been very limited [23,24]. GC−MS analysis requires derivatization (e.g., silylation) to convert PAT to a volatile compound. Although high sensitivity and fair selectivity may be obtained by HPLC−MS/MS, the equipment is relatively expensive and preferably uses an isotope internal standard, which is extremely expensive (^13^C_5–7_ patulin, 25 μg/mL × 1.2 mL, 6500.00 CNY = $1000) for patulin [33]. Thus, LC−MS/MS is not feasible for analyzing large numbers of samples in all laboratories. The selection of the analytical procedure for PAT determination should depend on the matrix and the sensitivity demanded for its routine analysis [29]. When HPLC−UV can meet the analytical requirements, it is always selected [29,31,32,34] due to its low costs. 

Major matrix components in tea, such as the polyphenolic water-soluble pigments thearubigin and theaflavin and fat-soluble ones such as chlorophyll and carotenoids are different from those in fruits. As such matrix components may interfere with the determination of PAT and removal of pigments is required, e.g., by sample pretreatment methods such as LLE or SPE. Thus, to be able to analyze PAT in tea samples, first existing analytical sample clean-up and chromatographic methods for PAT in fruits have to be tested and, if needed, adapted for tea samples.

In this paper, both the analytical goal, i.e., development of an efficient method for PAT analysis in tea, and the food safety goal, i.e., is the occurrence of PAT in different tea samples an issue? are addressed. For this purpose, HPLC−UV and GC−MS combined with trimethylsilylation derivatization were compared for their suitability to determine PAT in tea products and for cross validation. Also, attention was given to a simple sample pretreatment method in combination with HPLC analysis. Next, 219 commercial tea products with different stages of fermentation and ageing were analyzed for their PAT content to arrive at a food safety conclusion regarding the role of PAT in Chinese teas. To our knowledge, never before such an exhaustive targeted chemical analysis of the mycotoxin patulin in seven different types of teas with multiple samples per type by two complementary analytical methods was undertaken. Results are presented herein.

## 2. Results and Discussion

### 2.1. Development of HPLC-DAD Method for Analysis of PAT in Tea

Although the average tea consumption is a modest 1.55 g/person/day in China, this will vary strongly from person to person. As heavy tea drinkers are most at risk, a safety factor of 20 was used corresponding with a consumption of 30 g tea per person per day. This amounts to about 4 L of strong tea (7.5 g/L) per person per day. In turn, this translates—with a maximum safe dose of 30 µg PAT/person/day—that any tea should not contain more than 1 µg PAT per g (1 ppm). To be able to detect such concentrations accurately without the risk of false-positives, an analytical safety factor of 20 was set in this study. In other words, if the targeted analytical method for PAT in tea is able to accurately and precisely determine 50 ng PAT per g of tea (50 ppb), concentrations potentially deleterious for health (>1000 ppb) will not pose a problem from an analytical point of view. 

For the sample pre-treatment of PAT in fruit matrixes, mostly liquid-liquid extraction (LLE), and matrix dispersion SPE (dSPE) have been used [9,25,29,31,32]. Since matrix components in tea differ from those in fruit matrixes, pre-treatment methods used for fruit matrixes analysis should be investigated regarding their suitability. LLE methods include an alkaline, e.g., sodium bicarbonate clean-up [25,34]. However, such clean-up procedures appear to degrade patulin since it is unstable under alkaline conditions [31]. dSPE methods always use “primary secondary amine” (PSA) sorbents or graphitized carbon black (GCB) to remove matrix interference components such as polyphenolic compounds [26,35]. However, the efficiency for removing pigments from tea has not been investigated. To evaluate the feasibility of LLE and dSPE in tea analysis, four pretreatment experiments were used with different kinds of tea spiked with PAT standard solution (50 ng/g level). However, in order to judge the results first a proper HPLC method had to be chosen. 

Although RP-HPLC−UV has been employed as a convenient method for the routine analysis of PAT [32,34], it has some limitations in specificity and retention. 5-Hydroxymethylfurfural (HMF), which may be formed during food processing (Maillard reaction) and storage, particularly at high temperatures, may interfere and affect the PAT quantification with HPLC−UV [38]. Hydrophilic compounds such as PAT are difficult to retain on C18 columns, therefore most methods use large percentages of water (≥90%) in the mobile phase [9,28,29,30]. In this experiment, an Atlantis^TM^ T3 column was used for the separation. An optimized particle pore size and C18 surface concentration of stationary phase enable reliable operation with 100% aqueous mobile phases needed for retaining highly polar analytes. 5-HMF (10.2 min) and PAT (13.9 min) are well separated on this column (Figure S1). There was no interference of 5-HMF to PAT. The sensitivity is fit-for-purpose with 5 ng of PAT injected (corresponding with 5 ng PAT per g of tea) giving a peak with a S/N level of 100.

To obtain retention of PAT as well as high sensitivity, 95% water was used in the mobile phase in combination with a relatively large injection volume of 100 μL. The sample solution must have a low organic solvent content as otherwise PAT is not retained on the column. Figure S2 shows chromatograms of PAT and a green tea sample in pure ACN. No usable chromatogram results. When the ACN content in the sample solution was ≤5% (*v*/*v*), the retention was not affected. The column temperature significantly affected the retention of PAT on the column. The retention time was 13.9, 13.7, 13.0, 12.5 and 10.5 min at 20, 24, 28, 35, and 40 °C, respectively. Therefore, the column temperature must be controlled to obtained reproducible separations.

Having established the HPLC−UV conditions, next the sample preparation step was investigated. In short, the four selected methods were as follows (see for full experimental details Section 3.3): (1) extraction of tea with ACN, addition of GCB to adsorb pigments, drying of supernatant, redissolving in H_2_O; (2) extraction of tea with ACN, addition of PSA to adsorb pigments, drying of supernatant, redissolving in H_2_O; (3) extraction of tea aqueous solution with ethyl acetate, and alkaline clean-up [31]; (4) ultrasonic extraction with ACN, drying, extraction with H_2_O, filtration. In all cases, final extracts were analyzed by HPLC−UV. For the removal of pigments, method (1) worked best, the sample solutions were colorless. After method (4), sample solutions were pale yellow-green. Method (2) and (3) gave strongly colored samples and largely failed to remove pigments. The recoveries of PAT of the four methods are listed in Table 1. Although method (4) was not the best for removing pigments, the recoveries of PAT in the different tea samples were the highest, i.e., all >95%. Therefore, sample pretreatment method (4) was selected.

**Figure 2:**
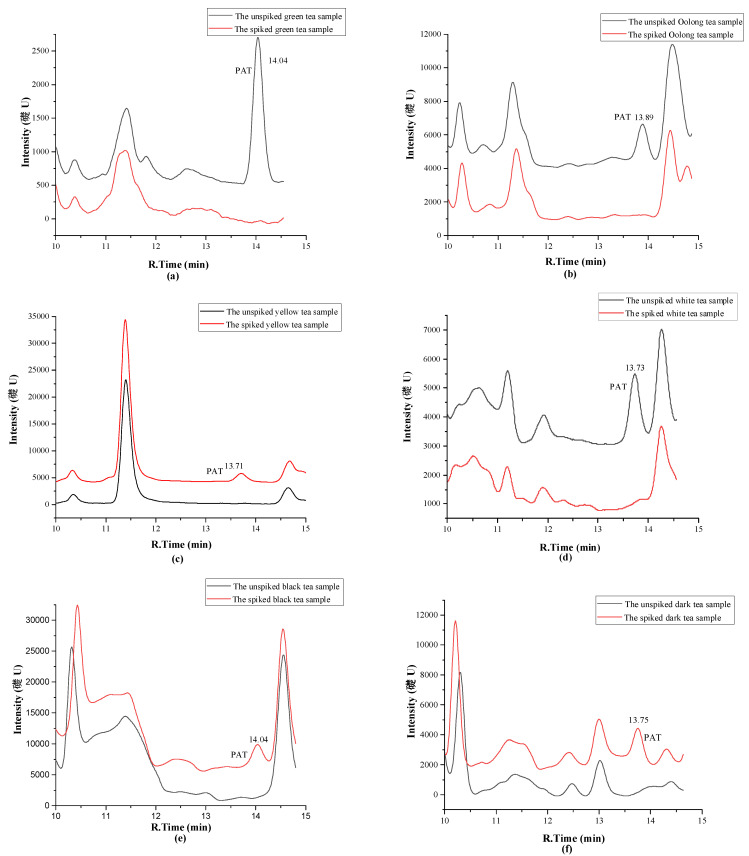

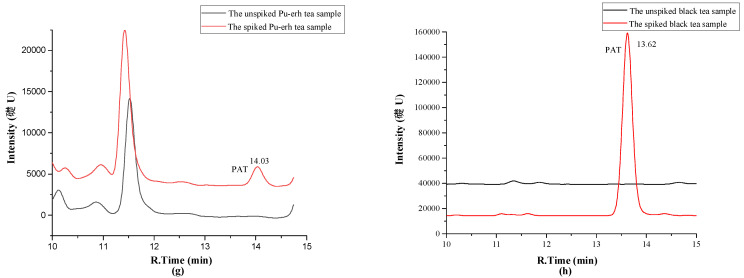
HPLC profiles of different blank teas and spiked teas (**a**–**g**: 50 ng/g; h: 5.0 µg/g). (**a**): green tea; (**b**): Oolong tea; (**c**): yellow tea; (**d**): white tea; (**e**): black tea; (**f**): dark tea; (**g**): Pu-erh tea; (**h**): black tea.

**Figure 3:**
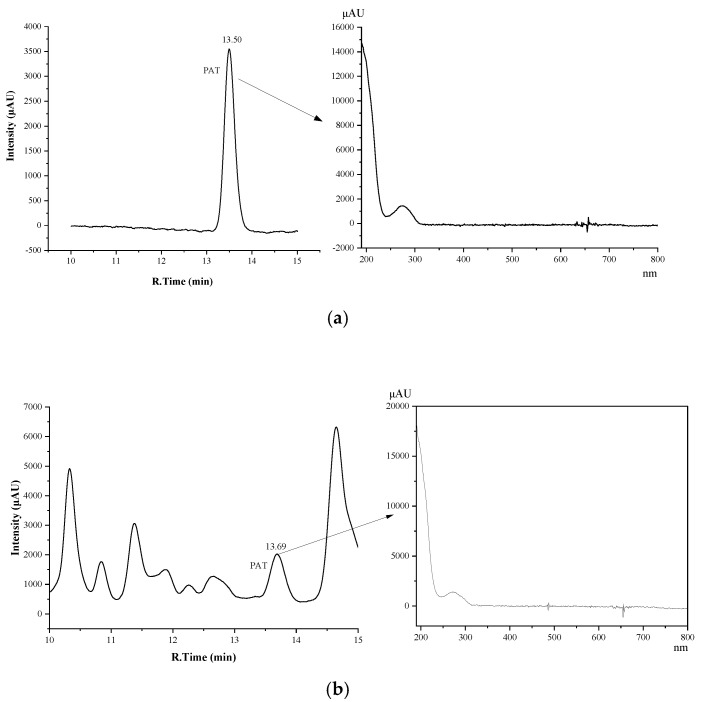
(**a**)**.** HPLC profile (left) and DAD−UV spectrum of PAT standard (50 ng/mL) (right). (**b**)**.** HPLC profile of completely fermented black tea #2 (Table S1) naturally contaminated with PAT (43.4 ng/g) (left) and DAD−UV spectrum of PAT peak (right).

All seven different types of tea were analyzed as such, and after a 50 ng/g spike with PAT, to determine the suitability of the HPLC−UV approach. The chromatograms of different blank tea samples and the same tea samples spiked with 50 ng/g PAT are shown in Figure 2a–h. Chromatograms and DAD−UV spectra of PAT standard (50 ng/mL) and a black tea sample naturally contaminated with PAT (43.4 ng/g) are shown in in Figure 3a,b. Again, a PAT concentration of 50 ng/g in tea is well below the PAT content of 1 µg/g that constitutes a health risk for heavy tea drinkers.

The results demonstrate that method (4) provides sufficient selectivity for the analysis of PAT in teas at the low concentration of 50 ng/g. Non-fermented green tea and partially fermented Oolong, yellow and white teas clearly contain fewer matrix components than the completely fermented black tea and dark tea but the PAT peak of tea spiked at only 50 ng/g is still well observable in the latter two teas. To show what a HPLC−UV profile of a potentially dangerous concentration of PAT looks like, in Figure 2h a 5.0 µg/g spike (5 ppm) of black tea is shown. Thus, the risk of false positive results appears very low. The characteristic absorption wavelength of the peak at retention time of PAT in a positive sample of black tea is the same as that of PAT standard (276 nm) (Figure 3). This further confirms the feasibility of the method for the analysis of PAT in tea.

### 2.2. On-Line Derivatization for GC−MS Analysis

To validate the HPLC−UV method, a second orthogonal analytical method, i.e., GC−MS, was used. GC−MS has higher selectivity due to its higher separation power and the ability to focus on a specific mass. Additionally, many higher MW matrix components that might interfere with PAT quantitation by HPLC−UV, will not elute from a GC even after silylation. For PAT analysis by GC−MS, both off-line TMS derivatization [25] and on-line N-methyl-N-(trimethylsilyl)trifluoroacetamide (MSTFA) derivatization (injection-port derivatization) [28] have been described. Injection-port derivatization saves sample preparation time. In this work, both derivatization methods were compared using in both cases BSTFA:TMCS (99:1) as silylating agent (Table 2).

The results show that there is no significant difference in derivatization efficiency between both methods when the injection-port temperature is 280 °C. The RSD of on-line derivatization was twice smaller than that of off-line derivatization. Thus, on-line derivatization was chosen in this study as it saves both time and effort besides being more precise.

**Figure 4:**
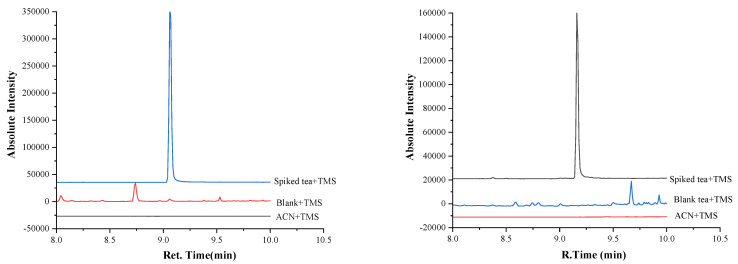
GC−MS SIM profiles at *m/z* 194 for NBA (left) and *m/z* 183 for PAT (right) of blank black tea spiked with 3-NBA (I.S.) and PAT, both at 5.0 ng/g.

For the quantitative analysis of PAT by GC−MS, different internal standards such as ^13^C_5–7_ patulin [27], hexachlorobenzene (HCB) [35], and 3-nitrobenzyl alcohol (3-NBA) [25] have been described in literature. Since ^13^C_5–7_ patulin is too expensive at $ 40,000/mg for routine use, and HCB might occur as organochlorine pesticide residue in tea, 3-NBA was selected as I.S. 3-NBA has never been detected in tea and is thus suitable to serve as an internal standard for GC−MS analysis of patulin in tea samples. Figure 4 shows the SIM (selected ion monitoring) profiles for 3-NBA and PAT added to blank black tea at 5 ng/g. Even at 5 ppb, the spike is well observable indicating a higher sensitivity and selectivity than that obtainable with HPLC−UV. Even in the corresponding TIC (total ion current) profile, the 5 ppb spikes are visible (Figure S3). Figure S4 shows the corresponding mass spectra of 3-NBA and PAT in Figure S3 (as TMS derivatives). Their mass spectra are identical with those shown in the literature [25,39]. Calibration curves were constructed by using as quantifying ion *m/z* 183 for PAT and *m/z* 194 for 3-NBA. The same ions were used in earlier GC−MS studies [25,39].

Sample pretreatment method (4) described in Section 2.1 was incompatible with GC−MS analysis due to residual water in the final sample solution. This water in the sample solution proved difficult to remove and seriously affected silylation efficiency. Thus, in case of GC−MS, tea was simply extracted by ACN followed by evaporation of the solvent. A disadvantage of having no pretreatment method was pollution of the injector with matrix constituents. After every 10 samples the injector liner and silica wool had to be replaced as otherwise a number of ghost peaks appeared. The GC−MS method without sample pretreatment can be used for analyzing PAT in tea, e.g., when no HPLC−UV is available. However, further research on sample pretreatment is advised when GC−MS is to be used for the routine analysis of PAT in tea samples.

### 2.3. Method Validation

**Figure 5:**
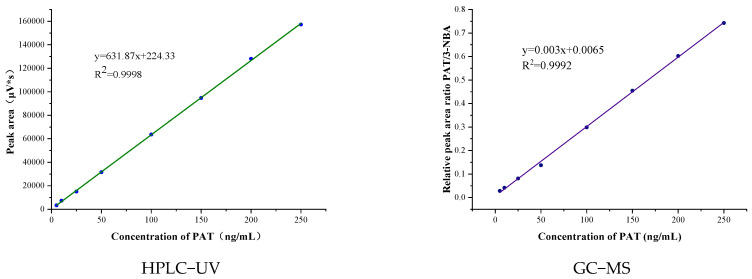
Calibration curves of HPLC−UV and GC−MS.

The calibration curves for HPLC−UV and GC−MS are shown in Figure 5 and exhibit excellent linearity over the entire concentration range. Limit of detection (LOD, S/N = 3) and limit of quantification (S/N = 10) in tea were 1.5 ng/g and 5.0 ng/g for HPLC, and 0.25 ng/g and 0.83 ng/g for GC−MS, respectively. The accuracy of both methods was determined by spiking four types of blank teas with different amounts of PAT and calculating the relative recovery. The precision was determined by calculating the SD of five replicates. Results are presented in Table 3.

The mean relative recovery of PAT was 89.7–102.6% in GC−MS and 95.2–102.5% in HPLC−UV, respectively. Relative standard deviations were 3.7–11.1% in case of GC−MS and 2.6–5.8% for HPLC−UV. So, the accuracy and precision of both methods meet the requirements for PAT analysis in tea samples.

### 2.4. Investigation of Patulin in Different Chinese Tea Products

To get a reliable impression regarding the occurrence of PAT in different tea products in China, 219 Chinese tea products with different degrees of fermentation and aging times were analyzed by HPLC−UV. Results are summarized in Table 4. Average PAT concentrations in all seven types of tea are low but in all groups variation is significant. Yellow tea has the lowest PAT content and black and dark tea the highest (Table 4). Ten samples with a high PAT content were also analyzed by GC−MS for confirmation and validation. The correspondence between both analytical methods was excellent (Table 5). Plotting the results against one another gives a straight line (r^2^ = 0.998) indicating that both—very different—methods provide a reliable outcome (Figure S5). Detailed results on all samples are listed in Table S1. Among the 219 samples, 10 tea products (4.5% of all samples) contained more than 10 ng/g of PAT, and only two tea products (1%) contained more than 50 ng/g PAT (Table 4). The highest PAT content of 124 ng/g was found in a dark tea sample. If 10 g of this tea product, corresponding to 2–5 L of tea, is consumed per person per day, a total of 1.2 µg/person/day of PAT is ingested, which is much lower than the maximum of 30 µg/person/day that is allowed for a 75 kg adult. Thus, based on this survey of 219 different tea products, one can conclude that tea products in China are safe with regard to their PAT content. No tea samples outside of China were analyzed so no definite conclusions regarding their safety can be made. However, in view of the large sample pool analyzed in this study, the low average PAT content and the wide safety margin for even the most contaminated sample, worldwide PAT contamination of tea is unlikely to constitute a safety risk to human health.

The 10 samples with more than 10 ng PAT per g tea (Table 5), include non-fermented, partially fermented, completely fermented and post-fermented products. There is no obvious correlation of PAT content with the degree of fermentation or aging. It has been reported that fermentation can cause an important reduction of PAT (>90%) due to yeast degradation [40,41]. Thus, fermentation of tea may help to reduce the PAT content of tea products. In 2016, Zhang et al. [24] reported that PAT was detected in 60% of raw Pu-erh (fermented tea, a kind of black tea) samples with a mean concentration of 1169 ng/g but in only 12.5% of the ripened Pu-erh samples at a mean concentration of 915 ng/g. However, none of the common PAT-producing fungi was detected in their samples. There were 17 Pu-erh samples among our 219 samples but their PAT content was low: average 1.2 ng/g, highest concentration 6.7 ng/g. The large discrepancy between our results and those reported by Zhang et al. needs to be further investigated. Most likely, it is related to the origin of the samples, their storage or processing. Another reason could be a false-positive result due to a lack of specificity of nominal LC−MS/MS analysis of low MW analytes such as patulin (MW = 154) in complex matrixes such as tea, i.e., risk of co-elution with a MW = 154 impurity in combination with using a common neutral loss such as 44 (*m/z* 153 → *m/z* 109). Duvivier et al. [42] have shown that triple-quadrupole MS exhibits lower specificity than high-resolution MS.

In conclusion, it can be stated that a large survey of different types of tea by two orthogonal targeted chemical-analytical methods showed that the mycotoxin patulin occurs in only a limited number of tea samples, and then always in low concentrations. In other words, even the consumption of many liters of strong tea does not lead to the ingestion of dangerous amounts of patulin. All 219 investigated Chinese teas were found to be safe with regard to their patulin content.

## 3. Materials and Methods

### 3.1. Materials, Reagents and Chemicals

Acetonitrile (ACN, HPLC-grade), ethyl acetate (analytical-grade) and other solvents were purchased from Merck (Darmstadt, Germany). Deionized water was obtained by using a Milli-QTM Direct ultrapure water system (Millipore, Boston, MA, USA). N,O-bis(trimethylsilyl)trifluoroacetamide (BSTFA) containing trimethylchlorosilane (TMCS) (99:1) was purchased from EKEAR (Shanghai, China). Primary secondary amine (PSA) sorbent and graphitized carbon black (GCB) were purchased from Macklin (Shanghai, China) and PAT (≥98%) and 3-NBA (≥98%), 5-HMF (≥98%) standards were purchased from Pribolab Biotech Co. (Tsingtao, China).

All 219 tea products were purchased in local markets and online e-shops. The tea products were divided into four categories: non-fermented, partially fermented, completely fermented and post-fermented. The sample information details are listed in Table S1.

### 3.2. HPLC Analysis

#### 3.2.1. Preparation of Standard Solutions

5.0 mg of patulin standard was dissolved in 10.00 mL of ACN and stored at –40 °C as the stock solution (500 μg/mL). The working solutions (5.00 μg/mL) were prepared by diluting the stock solution with H_2_O + ACN (95 + 5, *v*/*v*). PAT standard series were 5.00, 10.0, 25.0, 50.0, 100, 125 and 250 ng/mL in H_2_O + ACN (95 + 5, *v*/*v*) prepared by diluting the working solution.

#### 3.2.2. Preparation of Sample Solutions

1.00 g of tea powder was extracted three times with 2 mL of ACN in an acoustic wave bath for 10 min. After centrifugation (10,000 rpm for 10 min, GL-21M high speed centrifuge, Hunan Xiangyi Instrument Company, Changsha, China) of each extract, the three extracts were combined in a 10 mL glass tube. The extract was dried in stages under a N_2_ stream at 30 °C in a 2 mL tube. The residue was then extracted twice with 0.50 mL of H_2_O (1.00 mL in total). The extract was filtered through a 0.2 μm membrane (Millipore, Boston, MA, USA) and then analyzed by HPLC.

#### 3.2.3. HPLC Analysis

HPLC was performed using an HPLC series Nexera X2 equipped with a SPD-M20A DAD set at 276 nm and CTO 20A column oven (Shimadzu, Japan). Chromatographic separation was carried out with an Atlantis^TM^ T3 column (3 μm, 4.6 × 150 mm) (Waters Co. Milford, CT, USA). The column temperature was set at 20 °C. The injection volume was set at 100 μL and the flow rate at 0.8 mL/min. Solvent A was H_2_O and solvent B was acetonitrile (ACN). Gradient elution program was as follows: 0–13 min 5% B, 13–15 min 100% B, 15–20 min 5% B.

### 3.3. Extraction and Pretreatment Experiments

Four different pretreatment methods were tested using different kinds of tea spiked with PAT standard solution at a 50 ng/g level (10.0 μL of 5.0 μg/mL PAT was added to 1.00 g of tea powder):

(1) 1.00 g of tea sample was extracted by 2.0 mL of ACN for three times, 100 mg of GCB was added to the pooled ACN extracts to adsorb pigments, after centrifugation the ACN supernatant was dried by a N_2_ stream, the residue was redissolved in 1.00 mL of H_2_O, and the solution was analyzed by HPLC after filtration through a 0.22 μm membrane.
(2) 1.00 g of tea sample was extracted by 2.0 mL of ACN for three times, 100 mg of PSA was added to the pooled ACN extracts to adsorb pigments, after centrifugation the ACN supernatant was dried by a N_2_ stream, the residue was redissolved in 1.00 mL of H_2_O, and the solution was analyzed by HPLC after filtration through a 0.22 μm membrane.
(3) 1.00 g of tea sample was extracted by 10.0 mL of water for three times. After centrifugation (10,000 rpm for 10 min) of each extract, the three extracts were combined in a separatory funnel. The aqueous solution was extracted three times, each time with 15.0 mL of ethyl acetate. The combined organic solution was washed with 10.0 mL of 1.5% solution of sodium bicarbonate in water, and the aqueous phase was extracted with an additional portion of ethyl acetate (10.0 mL). The organic phases were combined, dried over anhydrous sodium sulfate, and evaporated to dryness in a stream of nitrogen. The residue was dissolved in 1.00 mL of water [30]. The solution was analyzed by HPLC after filtration through a 0.22 μm membrane.
(4) 1.00 g of tea sample was pretreated according to the procedure described in Section 3.2.2.

### 3.4. On-Line Derivatization for GC−MS Analysis

#### 3.4.1. Preparation of Standard Solutions

5.0 mg of patulin standard was dissolved in 10.0 mL of ACN and stored at –40 °C as the stock solution (500 μg/mL). The working solution (5.00 μg/mL) was prepared by diluting the stock solution. 5.0 mg of 3-NBA standard was dissolved in 10.0 mL of ACN and stored at –40 °C as the I.S. stock solution (500 μg/mL). The I.S. working solution (5.0 μg/mL) was prepared by diluting the stock solution. PAT standard series were 5.00, 10.0, 25.0, 50.0, 100, 150, 200 and 250 ng/mL in ACN and prepared by diluting the working solution. 200 μL of the standard series solution were transferred to autosampler vials by means of a 250 μL syringe. 2.0 μL of I.S. working solution and 25.0 μL of BSTFA (containing 1% TMCS) were added to the vial. After thorough mixing, 1.0 μL of the solution was injected into the GC−MS by the autosampler. The silylation derivatization was completed inside the injector of the GC. 

#### 3.4.2. Preparation of Sample Solutions

1.00 g of tea powder was extracted by ACN in an acoustic wave bath for 10 min. The extraction was repeated three times, each time with 2.0 mL of ACN. The extracts were combined in a 10 mL glass tube. The extract was dried under a N_2_ stream at 30 °C in a 2 mL autosampler vial in stages. The residue was then dissolved in 200 μL of ACN. 2.0 μL of I.S. working solution and 25.0 μL of BSTFA (containing 1% TMCS) were added to the solution. After thorough mixing, 1.0 μL of the solution was injected into the GC−MS by the autosampler.

#### 3.4.3. On-line Derivatization

On-line derivatization was used for comparison. For all of the tea products, sample preparation was as follows: (1) tea samples were pulverized by a grinder to tea powder; (2) 5.00 g of tea sample powder was weighed and added to a 50 mL fluoroethyleneproylene (FEP) centrifugation tube; (3) 10.0 mL of ACN was added to the tube; (4) the powder was ultrasonically extracted for 30 min; (5) vortex mixing for 30 s; (6) 2.00 mL of solution was filtered through a 0.22 μm membrane and transferred to a 5 mL polypropylene (PP) tube; (7) the tea extract solution was evaporated to dryness under a stream of N_2_. The derivatization was carried out after evaporation: first, 200 μL of ACN was added to the dry residue and vortexed for 1 min. Then 100 μL of the solution was transferred to a brown silanized magnetic screw-cap vial and 25.0 μL of BSTFA (containing 1% TMCS) were added. Each mixture was vortexed for 10 s, and heated at 50 °C for 40 min on a dry heating block. After cooling, 1.0 μL of liquid sample was injected into the GC−MS instrument for analysis.

#### 3.4.4. GC−MS Analysis

GC−MS analyses were carried out on a GC 2010 Plus coupled with a QP 2010 Ultra mass spectrometer and an AOC-20i autosampler (Shimadzu, Japan). An Rtx^®^-5 capillary column (30 m × 0.25 mm ID × 0.25 μm film thickness) (Restek, PA, USA) was employed. Carrier gas was helium at a constant column flow of 1.0 mL/min. Split ratio was 1:1 and injector temperature was set at 300 °C. The column oven temperature program was as follows: 100 °C held for 1 min, increased to 180 °C at 10 °C/min, then increased to 280 °C at 30 °C/min, and finally held at 280 °C for 12.67 min. The MS interface temperature was set at 280 °C. The EI source temperature was set at 250 °C and ionization occurred at 70 eV. Quantification was carried out in SIM mode, with the following ions to monitor: *m/z* 183 (quantitative ion), *m/z* 226 (qualitative ion 1) and *m/z* 136 (qualitative ion 2) for PAT and *m/z* 194 (quantitative ion), *m/z* 164 (qualitative ion 1), and *m/z* 210 (qualitative ion 1) for 3-NBA. A Mass spectrometric library (Version NIST11, Gaithersburg, MD, USA) was used for identifying the analyte and internal standard.

### 3.5. Method Validation

Specificity, linearity, Accuracy and Precision of the HPLC-DAD method were assessed with different tea samples (Non-Fermented (Green Tea), Partially Fermented (Oolong Tea), Completely Fermented (Black Tea), and Post-Fermented (Dark Tea)) Spiked at 10, 50 and 100 ng/g. Uncontaminated (Blank) Dried Tea Powder was Split Into 1.00 g Portions, which Were Spiked with 2.0 μL, 10 μL, and 20 μL of PAT Working Solution Patulin Solution (5.00 μg/mL in ACN) to Give Final Levels. The Samples Were Stored Overnight in the Dark at 4 °C Prior to Extraction

Linearity, accuracy and precision were assessed by carrying out five intra-day injections at each of the spiked concentrations. The standard curve was constructed by plotting peak areas versus concentrations and was analyzed using linear regression.

The Specificity, Linearity, Accuracy and Precision Were Determined with Tea Samples Spiked at 2.0, 10 and 20 ng/g (1.00 g Portions which Were Spiked with 2.0 μL, 10 μL, and 20 μL of PAT Working Solution Patulin Solution Diluted 5 Times (1.00 μg/mL in ACN) Including the Same Samples as Used for HPLC-DAD. Linearity, Accuracy and Precision Were Assessed by Carrying Out Five Intra-Day Injections at Each of the Spiked Concentrations. The Standard Curve Was Constructed by Plotting Ratios of PAT Peak Areas and I.S. Peak Areas Versus Concentrations and Was Analyzed Using Linear Regression

The LOD was calculated at an S/N ratio of 3.3 at 276 nm in HPLC−UV and at *m/z* 183 in GC−MS for PAT. Black tea spiked at 50 ng/g was pretreated according to the procedure described in Section 3.2 and Section 3.3. The obtained sample solution was then gradually diluted to get the concentration corresponding with an S/N of 3.3. 

## Figures and Tables

**Table 1:** Recovery of the four sample pre-treatment methods for PAT (*n* = 5).

Sample	Recovery (%)			
	Method 1	Method 2	Method 3	Method 4
Green tea	0.0	52.8 ± 10.6	67.0 ± 11.8	97.6 ± 5.7
Oolong tea	0.0	44.2 ± 9.5	55.4 ± 9.6	96.5 ± 4.7
Black tea	0.0	40.5 ± 11.8	65.4 ± 10.3	99.1 ± 4.1
Dark tea	0.0	43.9 ± 11.2	62.4 ± 7.8	96.5 ± 4.1

**Table 2:** Comparison of efficiency of on-line and off-line derivatization of 50 ng/mL PAT in ACN (*n* = 5) prior to GC−MS.

**On-line**	**Injector Temp. (°C)**	150	200	250	280	300
	**Peak area (SIM *m/z* 183)**	0	46.4 × 10^3^ ± 1.8 × 10^3^	52.0 × 10^3^ ± 1.3 × 10^3^	57.7 × 10^3^ ± 0.6 × 10^3^	57.8 × 10^3^ ± 0.7 × 10^3^
**Off-line**	**Heating time at 80 °C (min) (Inj. Temp. 280 °C)**	10	20	30	40	50
	**Peak area (SIM *m/z* 183)**	57.9 × 10^3^ ± 1.2 × 10^3^	57.4 × 10^3^ ± 1.1 × 10^3^	55.4 × 10^3^ ± 1.2 × 10^3^	52.3 × 10^3^ ± 1.7 × 10^3^	52.1 × 10^3^ ± 1.8 × 10^3^

**Table 3:** Accuracy expressed as % recovery of spiked samples and precision (*n* = 5) expressed as standard deviation of GC−MS and HPLC−UV methods.

Sample	Conc. (ng/g)	Recovery (%)	
		GC−MS	HPLC−UV
Green tea	10	89.7 ± 10.5	95.2 ± 4.6
	50	96.9 ± 8.4	97.6 ± 5.7
	100	94.3 ± 5.2	98.1 ± 3.4
Oolong tea	10	92.1 ± 3.4	97.4 ± 3.8
	50	94.9 ± 7.5	96.5 ± 4.7
	100	98.6 ± 5.1	101.2 ± 4.4
Black tea	10	102.6 ± 8.7	95.5 ± 3.6
	50	99.2 ± 7.3	99.1 ± 4.1
	100	92.4 ± 4.4	98.7 ± 4.4
Dark tea	10	95.6 ± 5.7	96.8 ± 3.3
	50	89.9 ± 8.2	96.5 ± 4.1
	100	93.3 ± 6.1	102.5 ± 2.7

**Table 4:** Average, minimal and maximum PAT content of different types of tea.

	Nr of Samples	Average PAT Concentration (ng/g)	Standard Deviation	Minimum and Maximum Conc.
Green tea	88	1.3	3.3	ND-22.8
Yellow tea	7	0.41	1.0	ND-2.9
White tea	13	3.3	3.6	ND-10.9
Oolong tea	21	1.5	4.7	ND-21.4
Pu-erh tea	17	1.2	2.2	ND-6.7
Dark tea	38	4.6	20.1	ND-124
Black tea	35	6.6	14.2	ND-68.5

ND, non-detectable, i.e., <LOD.

**Table 5:** Results of the 10 tea products with a PAT concentration higher than 10 ng/g.

No.	Sample Name	Fermentation Degree	Starting Year of Aging	Region	Place of Origin	Content (ng/g) (HPLC−UV/GC−MS)
2	Black tea	Completely fermented (Jingjunmei)	2017	East China	Fujian	43.4/42.2
4	Black tea	Completely fermented	2018	East China	Fujian	68.5/64.2
6	Black tea	Completely fermented	2016	East China	Fujian	11.2/12.3
7	Black tea	Completely fermented (Zhenshanxiaozhong)	2017	East China	Fujian	39.6/37.4
37	Green tea	Non-fermented	2018	Central China	Hunan	13.8/11.2
38	Green tea	Non-fermented (YYS008)	2021	Central China	Hunan	22.8/23.8
125	Oolong tea	Partially fermented	2018	East China	Fujian	21.4/20.7
127	White tea	Partially fermented	2010	East China	Fujian	10.9/10.4
165	Dark tea	Post-fermented (YYS024)	2021	Central China	Hunan	26.6/24.1
166	Dark tea	Post-fermented (YYS025)	2021	Central China	Hunan	124/112

## Data Availability

The data presented in this study are available in this article.

## Supplementary Materials

The following supporting information can be downloaded at: https://www.mdpi.com/article/10.3390/molecules27092852/s1, Table S1: detailed information regarding the 219 Chinese tea samples, including sample name, fermentation degree, starting year of aging, region, place of origin and concentration of PAT. Figures S1–S5: supplemental chromatograms, mass spectra and additional figures.

## Sample Availability

Not available.
