# Supplementary material for: Chromatographic Determination of the Mycotoxin Patulin in 219 Chinese Tea Samples and Implications for Human Health
Source: Molecules. 2022 Apr 29;27(9):2852. doi: 10.3390/molecules27092852 (PMC9103431; doi:10.3390/molecules27092852)
Supplement: Supplementary file 1 [file molecules-27-02852-s001.zip › molecules-1683162-supplementary.pdf]

Supporting information

Chromatographic determination of the mycotoxin patulin in 219 Chinese tea samples and implications for human health

Hai Li [1,a], Candi Liu [1,a], Shurong Luo [1], Sijie Zhu [1], Shan Tang [1], Huimei Zeng [1], Yu Qing [1], Ming Ma [1], Dong Zeng [2,*], Teris A. van Beek [4,*], Hui Wang [3], Bo Chen [1,*]

[1]Key Laboratory of Phytochemical R&D of Hunan Province and Key Laboratory of Chemical Biology & Traditional Chinese Medicine Research of Ministry of Education, Hunan Normal University, Changsha, 410081, China

[2]Hunan Provincial Center for Disease Control and Prevention, Changsha 410005, China

[3]Laboratory of Organic Chemistry, Wageningen University, Stippeneng 4, 6708 WE Wageningen, The Netherlands

[4]Changsha Institute for Food and Drug Control& National quality supervision and inspection center of liquor products (Hunan), Changsha 410013, China

Table S1

The detail information of 219 tea samples, including sample name, fermentation degree, started year of aging, region, place of origin and concentration of patulin.

No.	Sample name	Fermentation degree	Started year of aging	Region	Place of origin	Content (μg /kg）
1	Black tea	Completely fermented（Jingmaohou）	2019	Central China	Hunan	< LOD
2	Black tea	Completely fermented（Jingjunmei）	2017	East China	Fujian	43.36
3	Black tea	Completely fermented	2021	East China	Fujian	< LOD
4	Black tea	Completely fermented	2018	East China	Fujian	68.49
5	Black tea	Completely fermented（Wushangoncha）	2019	Central China	Hunan	< LOD
6	Black tea	Completely fermented	2016	East China	Fujian	11.23
7	Black tea	Completely fermented（Zhenshanxiaozhong）	2017	East China	Fujian	39.59
8	Black tea	Completely fermented	2016	Central China	Hunan	7.28
9	Black tea	Completely fermented	2017	East China	Fujian	6.43
10	Black tea	Completely fermented	2018	East China	Fujian	6.30
11	Black tea	Completely fermented	2017	Central China	Hunan	6.23
12	Black tea	Completely fermented	2019	Southwest China	Yunnan	5.79
13	Black tea	Completely fermented	2018	Central China	Hunan	5.71
14	Black tea	Completely fermented	2012	Southwest China	Yunnan	4.66
15	Black tea	Completely fermented	2019	Central China	Hunan	4.45
16	Black tea	Completely fermented	2018	East China	Fujian	3.89
17	Black tea	Completely fermented	2018	Abroad	India	3.75
18	Black tea	Completely fermented	2018	East China	Fujian	3.57
19	Black tea	Completely fermented	2019	Central China	Hunan	3.11
20	Black tea	Completely fermented	2018	Central China	Hunan	2.96
21	Black tea	Completely fermented	2018	East China	Fujian	2.90

22	Black tea	Completely fermented	2018	East China	Fujian	2.23
23	Black tea	Completely fermented	2019	East China	Fujian	< LOD
24	Black tea	Completely fermented	2018	East China	Fujian	< LOD
25	Black tea	Completely fermented	2018	East China	Fujian	< LOD
26	Black tea	Completely fermented	2018	North China	Beijing	< LOD
27	Black tea	Completely fermented	2018	Abroad	India	< LOD
28	Black tea	Completely fermented	2018	East China	Fujian	< LOD
29	Black tea	Completely fermented	2017	East China	Fujian	< LOD
30	Black tea	Completely fermented	2017	Abroad	India	< LOD
31	Black tea	Completely fermented	2017	Central China	Hunan	< LOD
32	Black tea	Completely fermented	2019	East China	Fujian	< LOD
33	Black tea	Completely fermented	2018	Southwest China	Yunnan	< LOD
34	Black tea	Completely fermented	2018	East China	Fujian	< LOD
35	Black tea	Completely fermented	2017	East China	Fujian	< LOD
36	Green tea	Non-fermented （YYS006）	2021	Central China	Hunan	< LOD
37	Green tea	Non-fermented	2018	Central China	Hunan	13.84
38	Green tea	Non-fermented （YYS008）	2021	Central China	Hunan	22.84
39	Green tea	Non-fermented	2021	Central China	Hunan	9.57
40	Green tea	Non-fermented	2018	East China	Jiangsu	6.99
41	Green tea	Non-fermented	2018	Southwest China	Sichuan	5.67
42	Green tea	Non-fermented	2021	Central China	Hunan	5.31
43	Green tea	Non-fermented	2018	Central China	Hunan	5.28
44	Green tea	Non-fermented	2018	Central China	Hunan	5.24
45	Green tea	Non-fermented	2020	Central China	Hunan	5.08
46	Green tea	Non-fermented	2021	Central China	Hunan	4.11

47	Green tea	Non-fermented	2021	Central China	Hunan	4.10
48	Green tea	Non-fermented	2018	Central China	Hunan	4.03
49	Green tea	Non-fermented	2018	Central China	Hunan	3.95
50	Green tea	Non-fermented	2018	Central China	Hunan	3.49
51	Green tea	Non-fermented	2021	Central China	Hunan	2.89
52	Green tea	Non-fermented	2018	Central China	Hunan	2.70
53	Green tea	Non-fermented	2018	Central China	Hunan	2.62
54	Green tea	Non-fermented	2018	Central China	Hunan	2.43
55	Green tea	Non-fermented	2015	East China	Fujian	2.09
56	Green tea	Non-fermented	2017	Central China	Hunan	1.97
57	Green tea	Non-fermented	2017	Central China	Hunan	< LOD
58	Green tea	Non-fermented	2018	Central China	Hunan	< LOD
59	Green tea	Non-fermented	2021	Central China	Hunan	< LOD
60	Green tea	Non-fermented	2021	Central China	Hunan	< LOD
61	Green tea	Non-fermented	2021	Central China	Hunan	< LOD
62	Green tea	Non-fermented	2021	Central China	Hunan	< LOD
63	Green tea	Non-fermented	2021	Central China	Hunan	< LOD
64	Green tea	Non-fermented	2021	Central China	Hunan	< LOD
65	Green tea	Non-fermented	2021	East China	Zhejiang	< LOD
66	Green tea	Non-fermented	2021	Central China	Hunan	< LOD
67	Green tea	Non-fermented	2021	Southwest China	Sichuan	< LOD
68	Green tea	Non-fermented	2021	East China	Zhejiang	< LOD
69	Green tea	Non-fermented	2021	East China	Zhejiang	< LOD

70	Green tea	Non-fermented	2021	Southwest China	Sichuan	< LOD
71	Green tea	Non-fermented	2021	Central China	Hunan	< LOD
72	Green tea	Non-fermented	2021	Central China	Hunan	< LOD
73	Green tea	Non-fermented	2021	Central China	Henan	< LOD
74	Green tea	Non-fermented	2021	Central China	Hunan	< LOD
75	Green tea	Non-fermented	2021	South China	Guangxi	< LOD
76	Green tea	Non-fermented	2021	Southwest China	Guizhou	< LOD
77	Green tea	Non-fermented	2020	East China	Jiangsu	< LOD
78	Green tea	Non-fermented	2019	South China	Guangdong	< LOD
79	Green tea	Non-fermented	2019	Central China	Hunan	< LOD
80	Green tea	Non-fermented	2019	Central China	Hunan	< LOD
81	Green tea	Non-fermented	2019	Central China	Hunan	< LOD
82	Green tea	Non-fermented	2019	Central China	Hunan	< LOD
83	Green tea	Non-fermented	2019	Central China	Hunan	< LOD
84	Green tea	Non-fermented	2019	Central China	Hunan	< LOD
85	Green tea	Non-fermented	2018	East China	Jiangsu	< LOD
86	Green tea	Non-fermented	2018	Central China	Hunan	< LOD
87	Green tea	Non-fermented	2018	Central China	Hunan	< LOD
88	Green tea	Non-fermented	2018	Central China	Hunan	< LOD
89	Green tea	Non-fermented	2018	Southwest China	Sichuan	< LOD
90	Green tea	Non-fermented	2018	Central China	Hunan	< LOD
91	Green tea	Non-fermented	2018	Central China	Hunan	< LOD
92	Green tea	Non-fermented	2018	East China	Shandong	< LOD

93	Green tea	Non-fermented	2018	Central China	Hunan	< LOD
94	Green tea	Non-fermented	2018	Central China	Hunan	< LOD
95	Green tea	Non-fermented	2018	East China	Fujian	< LOD
96	Green tea	Non-fermented	2018	Central China	Hunan	< LOD
97	Green tea	Non-fermented	2018	Central China	Hunan	< LOD
98	Green tea	Non-fermented	2018	Central China	Hunan	< LOD
99	Green tea	Non-fermented	2018	Central China	Hunan	< LOD
100	Green tea	Non-fermented	2018	Central China	Hunan	< LOD
101	Green tea	Non-fermented	2018	Central China	Hunan	< LOD
102	Green tea	Non-fermented	2018	Central China	Hunan	< LOD
103	Green tea	Non-fermented	2018	Central China	Hunan	< LOD
104	Green tea	Non-fermented	2018	Central China	Hunan	< LOD
105	Green tea	Non-fermented	2017	Central China	Hunan	< LOD
106	Green tea	Non-fermented	2020	South China	Guangxi	< LOD
107	Green tea	Non-fermented	2019	Central China	Hunan	< LOD
108	Green tea	Non-fermented	2019	Central China	Hunan	< LOD
109	Green tea	Non-fermented	2019	Central China	Hunan	< LOD
110	Green tea	Non-fermented	2019	Central China	Hunan	< LOD
111	Green tea	Non-fermented	2019	Central China	Hunan	< LOD
112	Green tea	Non-fermented	2018	Central China	Hunan	< LOD
113	Green tea	Non-fermented	2018	Central China	Hunan	< LOD
114	Green tea	Non-fermented	2018	Central China	Hunan	< LOD

115	Green tea	Non-fermented	2018	Southwest China	Guizhou	< LOD
116	Green tea	Non-fermented	2018	Central China	Hunan	< LOD
117	Green tea	Non-fermented	2018	Central China	Hunan	< LOD
118	Green tea	Non-fermented	2018	Central China	Hunan	< LOD
119	Green tea	Non-fermented	2018	Central China	Hunan	< LOD
120	Green tea	Non-fermented	2018	Central China	Hunan	< LOD
121	Green tea	Non-fermented	2018	East China	Fujian	< LOD
122	Green tea	Non-fermented	2017	East China	Zhejiang	< LOD
123	Green tea	Non-fermented	2017	Central China	Hunan	< LOD
124	Oolong tea	Partially fermented	2018	East China	Fujian	< LOD
125	Oolong tea	Partially fermented	2018	East China	Fujian	21.43
126	Oolong tea	Partially fermented	2019	East China	Zhejiang	< LOD
127	White tea	Partially fermented	2010	East China	Fujian	10.92
128	White tea	Partially fermented	2006	East China	Fujian	7.62
129	White tea	Partially fermented	2018	Central China	Hunan	6.72
130	Oolong tea	Partially fermented	2018	East China	Fujian	6.50
131	White tea	Partially fermented	2017	East China	Fujian	5.90
132	White tea	Partially fermented	2016	East China	Fujian	5.08
133	White tea	Partially fermented	2015	East China	Fujian	4.30
134	Oolong tea	Partially fermented	2018	East China	Fujian	3.60
135	Yellow tea	Partially fermented	2018	East China	Anhui	2.88
136	White tea	Partially fermented	2018	East China	Zhejiang	2.41
137	White tea	Partially fermented	2018	Central China	Hunan	< LOD
138	Oolong tea	Partially fermented	2019	East China	Fujian	< LOD
139	White tea	Partially fermented	2019	East China	Fujian	< LOD
140	Oolong tea	Partially fermented	2018	East China	Jiangxi	< LOD

141	Oolong tea	Partially fermented	2018	East China	Fujian	< LOD
142	White tea	Partially fermented	2018	East China	Fujian	< LOD
143	Yellow tea	Partially fermented	2018	East China	Anhui	< LOD
144	Oolong tea	Partially fermented	2018	East China	Zhejiang	< LOD
145	Oolong tea	Partially fermented	2018	East China	Jiangsu	< LOD
146	Oolong tea	Partially fermented	2018	East China	Fujian	< LOD
147	Yellow tea	Partially fermented	2018	East China	Anhui	< LOD
148	Oolong tea	Partially fermented	2018	Central China	Hunan	< LOD
149	Yellow tea	Partially fermented	2018	East China	Anhui	< LOD
150	White tea	Partially fermented	2018	East China	Zhejiang	< LOD
151	Yellow tea	Partially fermented	2018	East China	Anhui	< LOD
152	White tea	Partially fermented	2018	East China	Fujian	< LOD
153	Yellow tea	Partially fermented	2017	East China	Anhui	< LOD
154	Oolong tea	Partially fermented	2017	Central China	Hunan	< LOD
155	Oolong tea	Partially fermented	2020	East China	Taiwan	< LOD
156	Oolong tea	Partially fermented	2020	East China	Fujian	< LOD
157	Oolong tea	Partially fermented	2019	East China	Fujian	< LOD
158	Oolong tea	Partially fermented	2018	East China	Fujian	< LOD
159	Oolong tea	Partially fermented	2018	East China	Fujian	< LOD
160	Yellow tea	Partially fermented	2018	Central China	Hubei	< LOD
161	Oolong tea	Partially fermented	2018	East China	Fujian	< LOD
162	Oolong tea	Partially fermented	2018	South China	Guangdong	< LOD
163	White tea	Partially fermented	2018	East China	Fujian	< LOD
164	Oolong tea	Partially fermented	2018	East China	Taiwan	< LOD
165	Dark tea	Post-fermented (YYS024)	2021	Central China	Hunan	26.55
166	Dark tea	Post-fermented (YYS025)	2021	Central China	Hunan	123.6
167	Dark tea	Post-fermented	2012	Central China	Hunan	< LOD

168	Pu-erh tea	Post-fermented	1990	Southwest China	Yunnan	6.68
169	Dark tea	Post-fermented	2007	Central China	Hunan	6.23
170	Dark tea	Post-fermented	2021	Central China	Hunan	6.21
171	Dark tea	Post-fermented	2016	Central China	Hunan	5.72
172	Pu-erh tea	Post-fermented	2016	Southwest China	Yunnan	5.42
173	Pu-erh tea	Post-fermented	2018	Southwest China	Yunnan	4.18
174	Dark tea	Post-fermented	2021	Central China	Hunan	4.14
175	Pu-erh tea	Post-fermented	2014	Southwest China	Yunnan	3.58
176	Dark tea	Post-fermented	2018	Central China	Hunan	2.44
177	Dark tea	Post-fermented	2018	Central China	Hunan	< LOD
178	Pu-erh tea	Post-fermented	2016	Southwest China	Yunnan	< LOD
179	Dark tea	Post-fermented	2021	Central China	Hunan	< LOD
180	Dark tea	Post-fermented	2021	Central China	Hunan	< LOD
181	Dark tea	Post-fermented	2021	Central China	Hunan	< LOD
182	Dark tea	Post-fermented	2021	Central China	Hunan	< LOD
183	Dark tea	Post-fermented	2021	Central China	Hunan	< LOD
184	Dark tea	Post-fermented	2021	Central China	Hunan	< LOD
185	Dark tea	Post-fermented	2021	Central China	Hunan	< LOD
186	Dark tea	Post-fermented	2021	Central China	Hunan	< LOD
187	Dark tea	Post-fermented	2019	Central China	Hunan	< LOD
188	Dark tea	Post-fermented	2019	Central China	Hunan	< LOD
189	Dark tea	Post-fermented	2019	Central China	Hunan	< LOD

190	Dark tea	Post-fermented	2019	Central China	Hunan	< LOD
191	Dark tea	Post-fermented	2018	Central China	Hunan	< LOD
192	Pu-erh tea	Post-fermented	2018	Southwest China	Yunnan	< LOD
193	Dark tea	Post-fermented	2018	Central China	Hunan	< LOD
194	Pu-erh tea	Post-fermented	2018	Southwest China	Yunnan	< LOD
195	Dark tea	Post-fermented	2018	Central China	Hunan	< LOD
196	Pu-erh tea	Post-fermented	2018	Southwest China	Yunnan	< LOD
197	Dark tea	Post-fermented	2017	Central China	Hunan	< LOD
198	Dark tea	Post-fermented	2017	Central China	Hunan	< LOD
199	Pu-erh tea	Post-fermented	2017	Central China	Hunan	< LOD
200	Dark tea	Post-fermented	2017	Central China	Hunan	< LOD
201	Dark tea	Post-fermented	2017	Central China	Hunan	< LOD
202	Pu-erh tea	Post-fermented	2016	Southwest China	Yunnan	< LOD
203	Dark tea	Post-fermented	2016	Central China	Hunan	< LOD
204	Pu-erh tea	Post-fermented	2016	Southwest China	Yunnan	< LOD
205	Dark tea	Post-fermented	2016	Central China	Hunan	< LOD
206	Dark tea	Post-fermented	2015	Central China	Hunan	< LOD
207	Dark tea	Post-fermented	2015	Central China	Hunan	< LOD
208	Pu-erh tea	Post-fermented	2014	Central China	Hunan	< LOD
209	Dark tea	Post-fermented	2013	Central China	Hunan	< LOD
210	Pu-erh tea	Post-fermented	2020	East China	Fujian	< LOD
211	Pu-erh tea	Post-fermented	2020	East China	Fujian	< LOD
212	Pu-erh tea	Post-fermented	2019	South	Guangdong	< LOD

				China		
213	Pu-erh tea	Post-fermented	2018	Southwest China	Yunnan	< LOD
214	Dark tea	Post-fermented	2017	Central China	Hunan	< LOD
215	Dark tea	Post-fermented	2012	Central China	Hunan	< LOD
216	Pu-erh tea	Post-fermented	2012	Southwest China	Yunnan	< LOD
217	Dark tea	Post-fermented	2012	Central China	Hunan	< LOD
218	Dark tea	Post-fermented	2010	Central China	Hunan	< LOD
219	Dark tea	Post-fermented	2009	Central China	Hunan	< LOD

Note: < LOD, below than limit of detection.

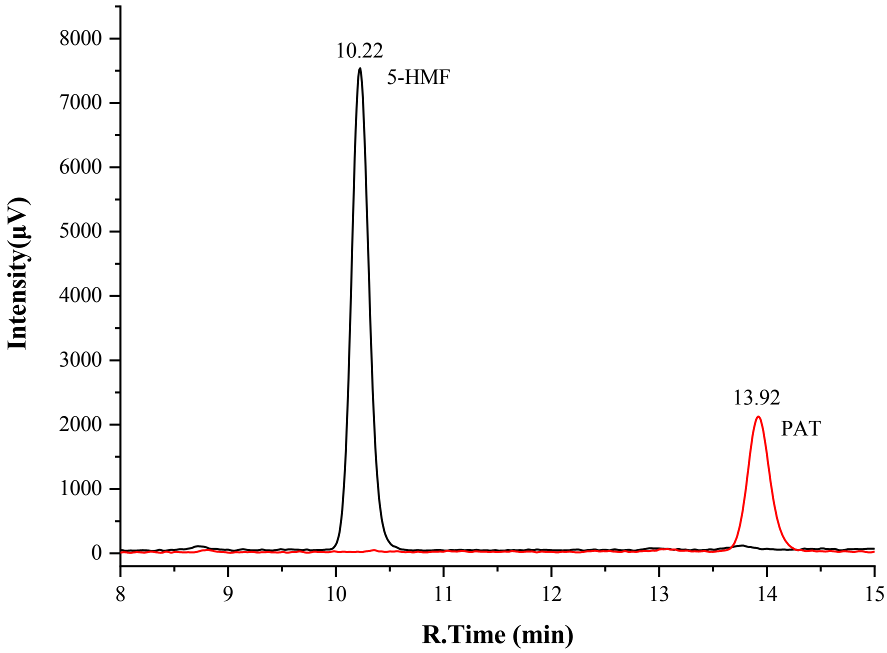

Figure S1. Separation of 5-HMF (1000 ng/mL) and PAT (50 ng/mL) on an Atlantis^{TM} T3 column (100 μL injection)

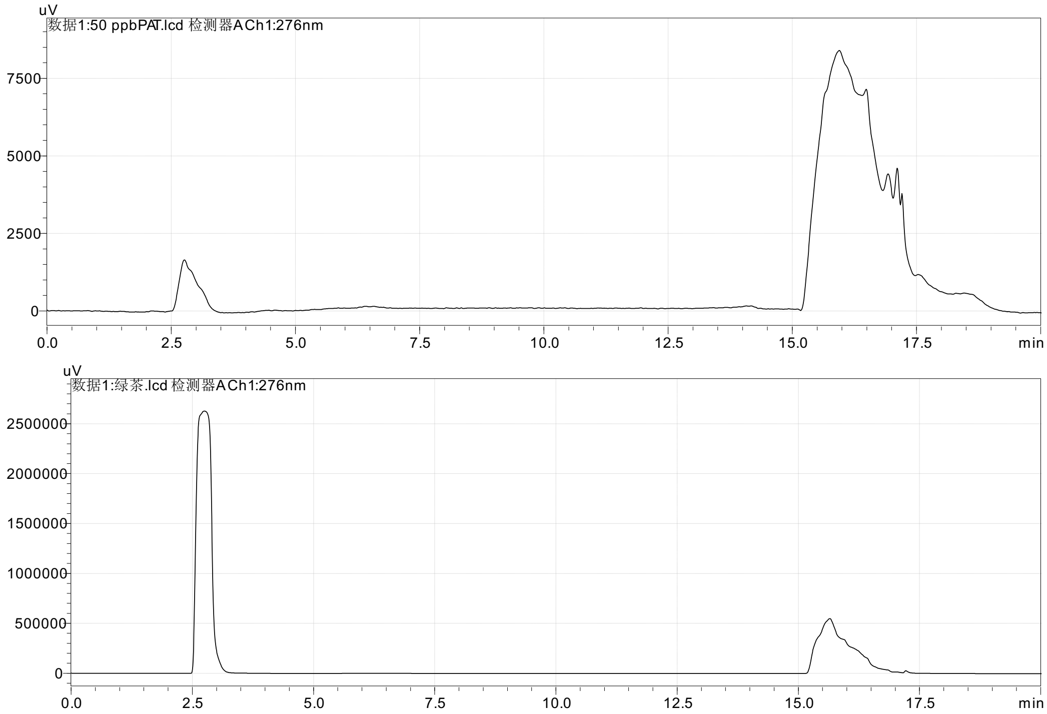

Figure S2. HPLC profiles of PAT (50 ng/mL) (upper) and green tea extracts (lower) in pure ACN (100 μL injection)

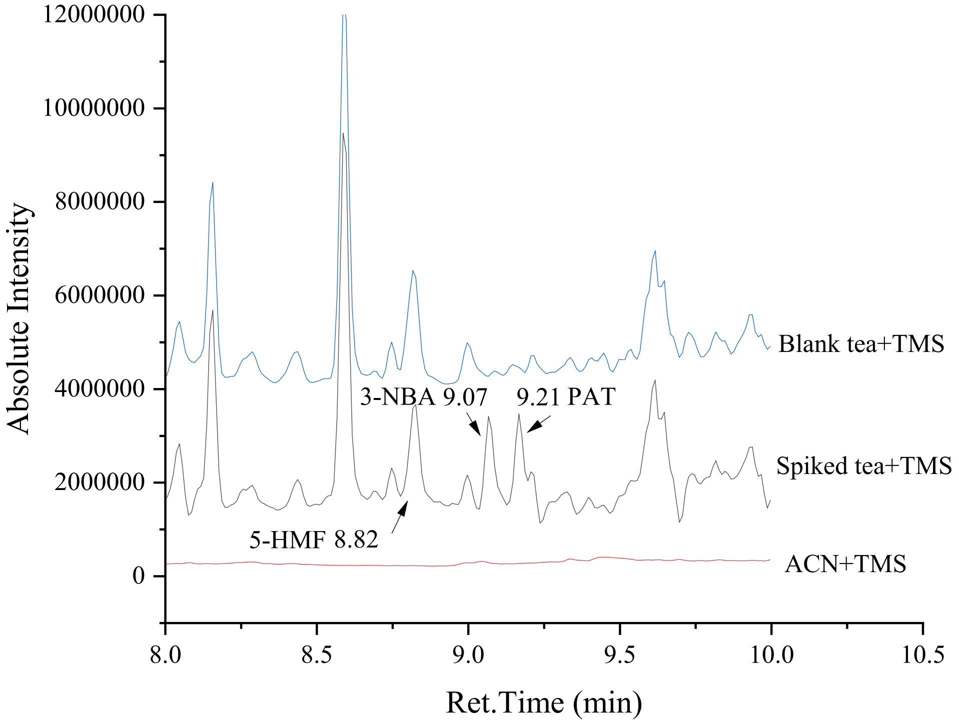

Figure S3. Total ion chromatogram of blank black tea spiked with 3-NBA (5.0 ng/g) and PAT (5.0 ng/g).

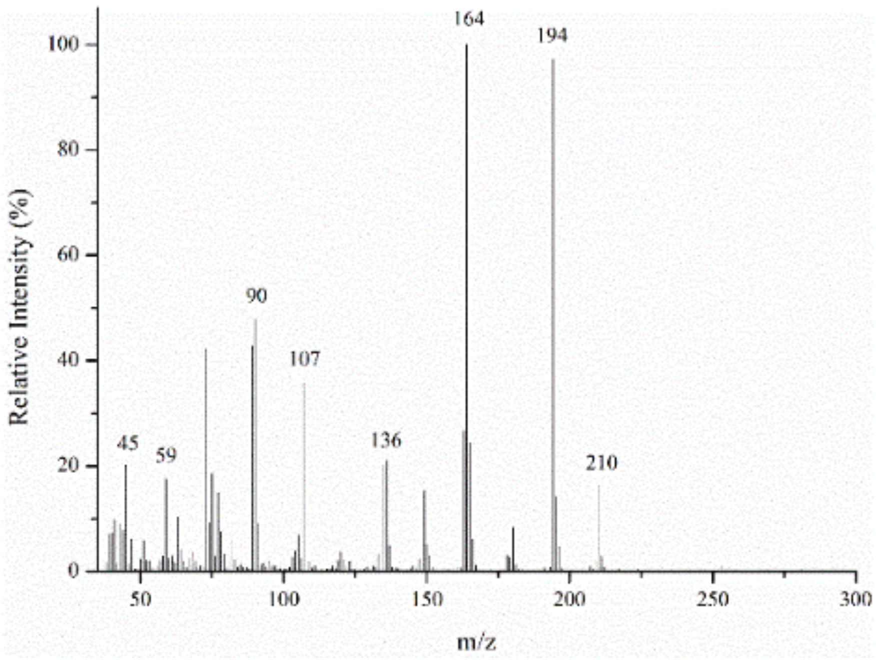

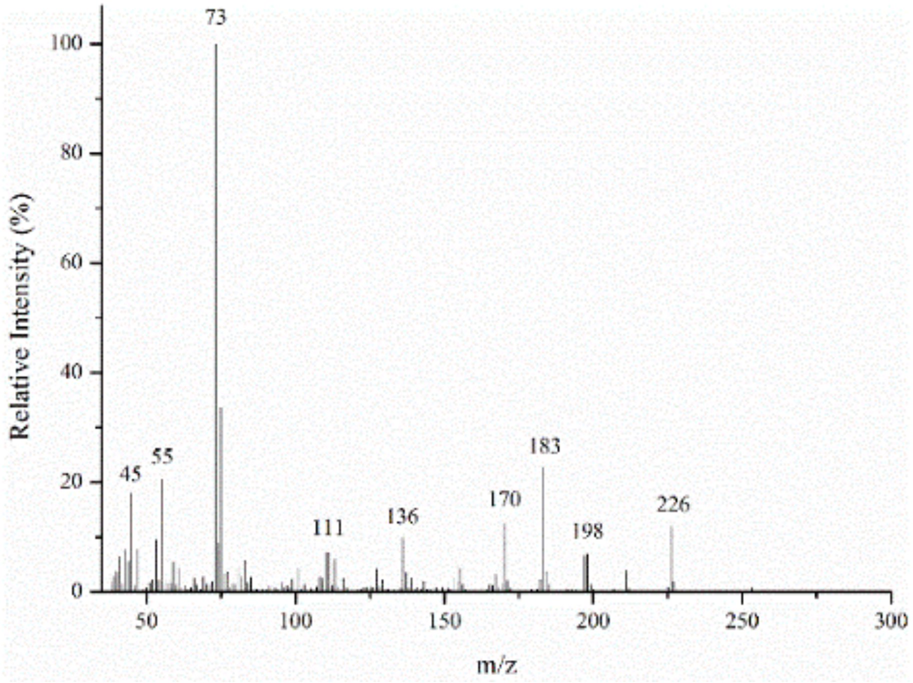

Figure S4. Mass spectra of 3-NBA (upper) and PAT (lower) (as TMS derivatives) taken from Fig. S3.

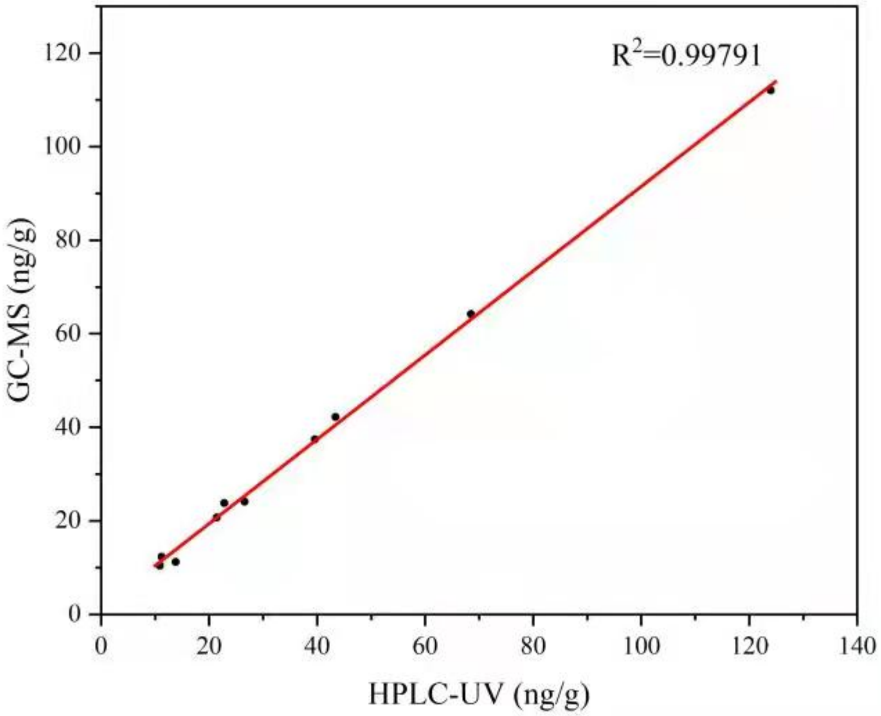

Figure S5. Correlation between HPLC-UV and GC-MS results for the 10 tea samples naturally contaminated with PAT and containing > 10 ng/g.
